# Effects of self-assembled cell-penetrating peptides and their nano-complexes on ABCB1 expression and activity

**DOI:** 10.22038/ijbms.2021.51675.11727

**Published:** 2021-03

**Authors:** Mehri Niazi, Parvin Zakeri-Milani, Mehdi Soleymani-Goloujeh, Ali Mohammadi, Muhammad Sarfraz, Raimar Löbenberg, Saeedeh Najafi-Hajivar, Javid Shahbazi-Mojarrad, Masoud Farshbaf, Hadi Valizadeh

**Affiliations:** 1 Student Research Committee, Faculty of Advanced Medical Sciences, Tabriz University of Medical Sciences, Tabriz, Iran; 2 Drug Applied Research Center and Faculty of Pharmacy, Tabriz University of Medical Sciences, Tabriz, Iran; 3 Liver and Gastrointestinal Diseases Research Center and Faculty of Pharmacy, Tabriz University of Medical Sciences, Tabriz, Iran; 4 Department of Stem Cells and Developmental Biology, Cell Science Research Center, Royan Institute for Stem Cell Biology and Technology, ACECR, Tehran, Iran; 5 Immunology Research Center, Tabriz University of Medical Sciences, Tabriz, Iran; 6 College of Pharmacy, Al Ain University of Science and Technology, Al Ain 64141, UAE; 7 Faculty of Pharmacy and Pharmaceutical Sciences, Katz Group Centre for Pharmacy and Health Research, University of Alberta, Edmonton, Alberta, T6G 2H5, Canada; 8 Biotechnology Research Center and Faculty of Pharmacy, Tabriz University of Medical Sciences, Tabriz, Iran

**Keywords:** Cancer therapy, CPPs, Doxorubicin, Multi-drug resistance, P-gp

## Abstract

**Objective(s):**

Doxorubicin (Dox) is one of the most well-known chemotherapeutics that are commonly applied for a wide range of cancer treatments. However, in most cases, efflux pumps like P-glycoprotein (P-gp), expel the taken drugs out of the cell and decrease the Dox bioavailability. Expression of P-gp is associated with elevated mRNA expression of the ATP-binding cassette B1 (ABCB1) gene.

**Materials and Methods:**

In the current study, different sequences of cell-penetrating peptides (CPPs) containing tryptophan, lysine, and arginine and their nano-complexes were synthesized and their impact on the expression and activity of the ABCB1 gene was evaluated in the A549 lung carcinoma cell line. Furthermore, the cellular uptake of designed CPPs in the A549 cell line was assessed.

**Results:**

The designed peptides, including [W4K4], [WR]3-QGR, R10, and K10 increased Dox cytotoxicity after 48 hr. Furthermore, arginine-rich peptides showed higher cellular uptake. Rhodamin123 accumulation studies illustrated that all the obtained peptides could successfully inhibit the P-gp pump. The designed peptides inhibited the ABCB1 gene expression, of which, [W4K4] resulted in the lowest expression ratio.

**Conclusion:**

[W4K4], [WR]3-QGR, R10, and K10 could successfully increase the Dox cytotoxicity by decreasing the efflux pump gene expression.

## Introduction

Cancer, uncontrolled growth of abnormal cells, is considered a global crisis for the health community (1). In this regard, there has been a growing concern upon applying novel diagnostics and therapeutics to reduce its mortality (2, 3). Lung cancer is known as one of the most commonly occurring cancers in adults that leads to death in most cases (4). Although chemotherapy is an indispensable part of cancer treatment (5), two main impediments reduce its efficiency. First, multidrug resistance (MDR) which is the leading cause of most chemotherapy failures and second is the destructive side effects of chemotherapeutics as a result of their unselective entrance to both normal and tumor cells (6). MDR is a cellular hindrance towards chemotherapy in which tumor cells do not respond to therapeutic agents (7). A wide range of mechanisms is involved in MDR ranging from cellular factors to physiological conditions. In another view, this drug resistance could be based on cancer cell genetics and gradual response to chemotherapeutics over the period of treatment, which are called inherent and acquired responses, respectively (8, 9). Mild to harsh acidic environment, hypoxia, abnormal vascularization, as well as high interstitial fluid pressure are some of the physiological conditions related to MDR (10, 11). On the other hand, drug resistance occurs due to numerous cellular factors, including enhanced drug metabolism and detoxification, reduced bioactivation of drugs, disruption of apoptotic signaling pathways, and reduced influx of drugs, namely increased drug efflux (11-16). Activation of special efflux pumps (MDR proteins) such as P-glycoprotein (P-gp) across the membrane is recognized as a crucial cause for MDR (17-20). ATP-binding cassette (ABC) is one of the most studied MDR protein superfamilies that is physiologically responsible for transporting different substrates *via* special pumps across the membrane by ATP consumption against their concentration gradient (19, 21, 22). There are 48 genes related to ABC proteins which are mainly expressed in human cells. Based on their functions and structural similarities, ABC proteins are divided into seven subfamilies (ABC-A to ABC-G), three of which have strongly been approved to be responsible for MDR including MDR-associated protein (MRP1, ABCC1), breast cancer resistance protein (MXR or BCRP, ABCG2), and P-gp (MDR1, ABCB1) (23-25). P-gp is a single polypeptide comprising twelve transmembrane domains (TMDs) and two nucleotide-binding domains (NBDs) (26) that transports a wide range of compounds such as anticancer drugs (Doxorubicin, Paclitaxel, Vincristine, Bisantrene, etc.), HIV protease inhibitors (Ritonavir, Saquinavir, Nelfinavir), analgesics (Morphine), antihistamines (Terfenadine, Fexofenadine), natural products (Curcuminoids, Colchicine), antibiotics (Erythromycin, Gramicidin A), lipids, and peptides (24). P-gp is mainly expressed in all cells but is overexpressed in cancer cells (17). Doxorubicin (Dox, Adriamycin) is an antineoplastic or cytotoxic chemotherapeutic which is applied for treatment of different cancers. However, due to the robust drug resistance provided by cancer cells against Dox, its therapeutic efficacy is significantly limited (27). In the recent decade, a wide range of approaches like silencing MDR-related miRNAs, altering regimen of chemotherapeutics (combination therapy), applying monoclonal antibodies reversing P-gp, designing novel agents that are not recognized by P-gp, P-gp modulators, and innovative classes of nanometric drug delivery systems (DDSs) have been introduced and investigated to tackle drug resistance and improve chemotherapy efficiency (7, 17, 28, 29). It has been proposed that amphiphilic, cationic molecules could inhibit negatively charged P-gp efflux pump (30). Therefore Cell-penetrating peptides (CPPs) have gained much attention due to their ability to overcome P-gp related drug resistance. CPPs are nano-scale short sequences of amino acids with cationic or amphiphilic features (31, 32). CPPs are able to deliver different kinds of cargoes into various cells in a nontoxic manner (33, 34). In this study, for the first time, ABCB1 expression and P-gp activity on Dox-resistant A549 lung cancer cell line were investigated in the presence of amphiphilic-cationic CPPs and their nano-complexes. 

## Materials and Methods

*Materials*

O-(Benzotriazole-1-yl)-N,N,N,N′-tetramethyluronium tetrafluoroborate (TBTU), hydroxybenzotriazole (HOBt), trifluoroacetic acid (TFA), 5(6)-carboxyfluorescein (FAM), triisopropylsilane (TIPS), phenol, piperidine, ethanol (95%), isopropanol, trizol, and chloroform were obtained from Sigma-Aldrich (Sigma, Saint, Louis, USA). N-ethyldiisopropylamine (DIPEA) was acquired from Merck (Merck, Germany). Dimethylformamide (DMF) and dichloromethane (DCM) were purchased from Scharlau Chemie (Barcelona, Spain). Fluorenylmethyloxycarbonyl (Fmoc)-amino acid derivatives and 2-chlorotrityl chloride resin (capacity: 1.08 mmol g^−1^) were purchased from AAPPTec (Louisville, KY, USA). RPMI-1640 medium and fetal bovine serum (FBS) were purchased from Gibco (Grand Island, NY, USA), Penicillin/streptomycin solution was provided by Applichem (Darmstadt, Germany). 3-(4,5-dimethyl-2-thiazolyl)-2,5-diphenyl-2H-tetrazolium bromide (MTT) was obtained from Roth (Roth, Germany). Trypsin-EDTA (ethylenediaminetetraacetic acid) was obtained from Invitrogen (Invitrogen, Carlsbad, USA). Cell culture T-75 flasks and 6- and 96-well plates were obtained from Biofil (Canada) and SORFA (China). A549 cell line was provided by the Pasteur Institute (Tehran, Iran). 

*Synthesis of CPP*

Peptides were manually synthesized by the solid-phase peptide synthesis (SPPS) method on 2-chlorotrityl chloride resin in glass reaction vessels (35, 36). Eleven different sequences were synthesized, and their nano-complexes were prepared (Table 2). Briefly, CPPs were synthesized by swelling 250 mg of resin in 3 ml dry DCM for 1 hr under dry nitrogen. Then, the DCM was drained from resin and Fmoc-protected amino acid was dissolved in dry DMF and poured into the reaction vessel, and shaken to be coupled to the resin in the presence of DIPEA for almost three hours. The coupling process was monitored by the ninhydrin test. In this step, other reacting trityl groups were end-capped using 0.2 ml ethanol and shacking for 15 min. The ethanol was filtered and washed by DCM (2×2 ml), DMF (2×2 ml), and methanol (2×2 ml). After coupling of the first amino acid, its FMOC group was deprotected by adding piperidine/DMF solution (20% v/v, 2 ml) and shaking for 30 min under argon atmosphere. The Ninhydrin test was carried out to monitor the deprotection process. Other amino acids were also coupled to the peptide chains in the presence of appropriate amounts of TBTU, HOBt, and DIPEA and in a repeated cycle of deprotection, washing, coupling, and washing. Peptide derivatives were cleaved and deprotected by adding 10 ml of cleavage cocktail (TFA/H_2_O/Phenol/TIPS: 88/5/5/2 v/v/v/v). The reaction vessel was then stirred for three hours. Then the volume of filtrates was reduced to one-third of the initial volume using a rotary-evaporator. The peptides precipitated by addition of cold diethyl ether (10 times more than the cocktail volume). Finally, the peptides were centrifuged at 1792 g for 4 min, separated, dried, and stored in Eppendorf tubes at -20 °C. 

*Preparation of CPP/E*_10_ *nano-complexes*

Nano-complexes were prepared in the presence of positively charged amino acids of synthesized peptides and negatively charged E_10_. Peptides were dissolved in dimethyl sulfoxide (DMSO) and E_10_ was then added in 1:5 ratio (E_10_: CPP) to form the nano-complexes.

*Characterization techniques*

The mean hydrodynamic diameter and size distribution of the chosen peptide (P3) and its nano-complexes (P3/E_10_) were evaluated by a Nano S Zetasizer (Malvern Instruments, Malvern, UK). The sample was diluted in distilled water at pH = 7.4 and the analyses were performed at 25 °C and repeated three times per sample. Scanning Electron Microscopy (SEM) was applied to determine the surface morphology, average diameter, and size of nano-complex. The day before the examination, the sample was prepared by drop-casting of a 5 mM aqueous solution (20 µl) dissolved in DMSO onto aluminum foils. It was coated with gold, lyophilized, and analyzed by SEM (Mira3, FEG-SEM, Tescan, USA, 5.0 kV) in a high vacuum mode.

*Cell culture*

A549 cell line is derived from a drug-resistant human lung carcinoma tissue and adherent in culture. This cell line was obtained from the Pasture Institute (Tehran, Iran). It was cultured in RPMI-1640 medium supplemented with 10% FBS, 100 units/ml penicillin, and 100 µg/ml streptomycin at 37 °C in 5% CO_2_ a humidified atmosphere. Moreover, to develop resistance, 0.1 µM Dox was added to the medium which was not toxic to the cells.

*Cell viability*

Cells were detached from flasks by trypsin and seeded in 96-well plates with a cell density of 1.5×10^4 ^cells/well (containing 200 μl/well of medium) and incubated for 24 hr at 37 °C in a humidified atmosphere of 5% CO_2_. The next day, cells were treated with different concentrations of peptides (15, 25, 50 μM), and incubated at the same condition for 24 hr. Afterward, Dox was added to plates with 20 μM concentration (IC_50_), and cells were incubated for the next 24, 48, 72 hr. The MTT assay was performed according to literature (37). Data were collected from at least 3 independent experiments performed in triplicates and analyzed using the Graphpad Prism 7 software package.

*Cellular uptake*

*Fluorescent Microscopy. *In order to study the cellular uptake of FAM-labeled peptides in the A549 cell line, cells were seeded in 6-well plates and incubated overnight. Then they were washed with PBS, treated with FAM-labeled peptides and nano-complexes, and incubated for 90 min at 37 °C. Next, the medium was removed and cells were washed with PBS three times. In order to fix the cells in 10 ml volume, 1 ml of formaldehyde (3.7%) was diluted with 9 ml PBS and then 2 ml of prepared fixative was added to cells. Cells were then washed with PBS three times. Samples were stored and protected from light at 4 °C until the coverslips were placed cell-side-down on cytoslides and observed using an Olympus IX81 fluorescence microscope (Olympus Optical Co., Tokyo, Japan).

*Flow cytometry*

First, cells were seeded in 6-well plates (3×10^5^ cells/well) and incubated for 24 hr. Then the medium was removed and cells were washed with PBS. FAM-labeled peptides and nano-complexes were added to wells and incubation was continued for 90 min. The old medium was removed and cells were washed with PBS three times and detached by trypsin/EDTA. Then 2 ml of medium was added to each well, and cells were centrifuged for 5 min, followed by washing three times with PBS and finally suspended in flow cytometry buffer to study the cellular uptake of FAM-labeled peptides and nano-complexes.

*Rhodamine123 accumulation*

In order to evaluate the penetrating effect of designed peptides on rhodamine123 accumulation (as P-gp substrate), the A549 cell line was seeded in 24-well plates and incubated for 24 hr. After removing the old medium, peptides were added to wells at a concentration of 50 µM and the incubation was continued for 48 hr. Then the peptide solution was removed and cells were washed three times with PBS. Then 0.5 µM rhodamine123 solution was added to each well, and cells were incubated for 3 hr. Finally, after washing with cold PBS, cells were lysed with Triton X-100 (1%) and centrifuged at 112 g for 5 min. The fluorescence was read by a spectrometer.

*ABCB1 expression assay*

*Real-time PCR*. To simultaneously monitor the PCR reaction in real-time and amount of products (DNA, cDNA, or RNA), cells were seeded in 6-well plates and kept overnight to adhere. Then, cells were treated with 50 µM of chosen peptides (P3, P5, P7, and P8) and their nano-complexes (P3/E_10_, P5/E_10_, P7/E_10_, and P8/E_10_) and incubated for 24 hr. Four different samples were prepared for assessment consisting of peptides (P3, P5, P7, and P8), peptide-Dox conjugates (P3-Dox, P5-Dox, P7-Dox, and P8-Dox), nano-complex (P5/E_10_), and nano-complex-Dox (P5/E_10_-Dox). Cells without any treatment were considered as control. On the third day of the experiment, Dox was added to the wells, and incubation was continued for the next 48 hr. Cells were washed twice by PBS, 100 µl trypsin/EDTA was added, and incubated for 5 min. After detachment, cells were transferred to Eppendorf tubes and washed twice by PBS. After addition of 1 ml Trizol, cells were shaken by vortex and incubated for 5 min at room temperature. Then 200 µl chloroform was added to each tube and they were shaken slowly for 15 sec, placed in ice for 5 min, and centrifuged for 15 min at 16128 g, 4 °C. The supernatant was transferred to RNAase free tubes, isopropanol added, and incubated for 15 min at 4 °C. Samples were centrifuged for 15 min at 16128 g, 4 °C. After discarding the supernatant, 1 ml of cold ethanol (75%) was added to samples and they were centrifuged for 10 min at 6300g, -20 °C. 50 µl diethyl pyrocarbonate (DEPC)-treated water was added to samples. For the synthesis of the first-strand cDNA from RNA, RevertAid^TM^(First-strand cDNA synthesis kit, Thermo-Fisher, Waltham, MA) was employed. To this end, 1 μl 5x reaction buffer, 1 μl RiboLock, and 1 μl dNTP were added to samples. Then 1 μl Reverse Transcriptase was added to the mixture and it was kept in PCR for 60 min at 42 °C**. **Reaction finished by increasing the temperature to 70 ^°^C for 10 min and the sample was transferred to ice. For approving complete synthesis of cDNA, electrophoresis was carried out. The ABCB1 expression level was also determined using the Corbett Rotor gene 6000 device and Takara Master Mix. Primers were designed and synthesized by Pishgam Company. Table 1 lists the primers and their properties. First, primers were centrifuged at 6300 g for 20 sec at 4 °C. Then DEPC-treated water was added to the diluted primers. Vials containing primers were kept at lab temperature for 30 min, centrifuged in the same condition, and kept at -20 °C.

Amplification was carried out using SYBR^®^ Premix Ex Taq™ II (RR820, USA) in a Rotor-Gene 6000 real-time PCR detection system (Corbett, UK), according to the manufacturer’s instructions. β-actin was used as a reference (housekeeping) gene to evaluate and compare the quality of different cDNA samples. Finally, before the data analysis, melting curves were assessed to prove the specific gene peak and absence of dimer primers. To achieve higher standards, the concentrations and T_m_ of primers were normalized. The synthesized cDNAs for all eleven samples were assessed to monitor the expression level of the ABCB1 gene.

## Results

*Synthesis and characterization*

Eleven sequences of peptides with different patterns were manually synthesized based on the SPPS method. The ninhydrin test was implemented to monitor the synthesis process and deprotection-coupling cycle. In order to obtain the peptides for the experiment, they were cleaved from resin, lyophilized, and kept at -20 ºC. Table 2 shows all the synthetic peptides and their properties.

**Figure 1:**
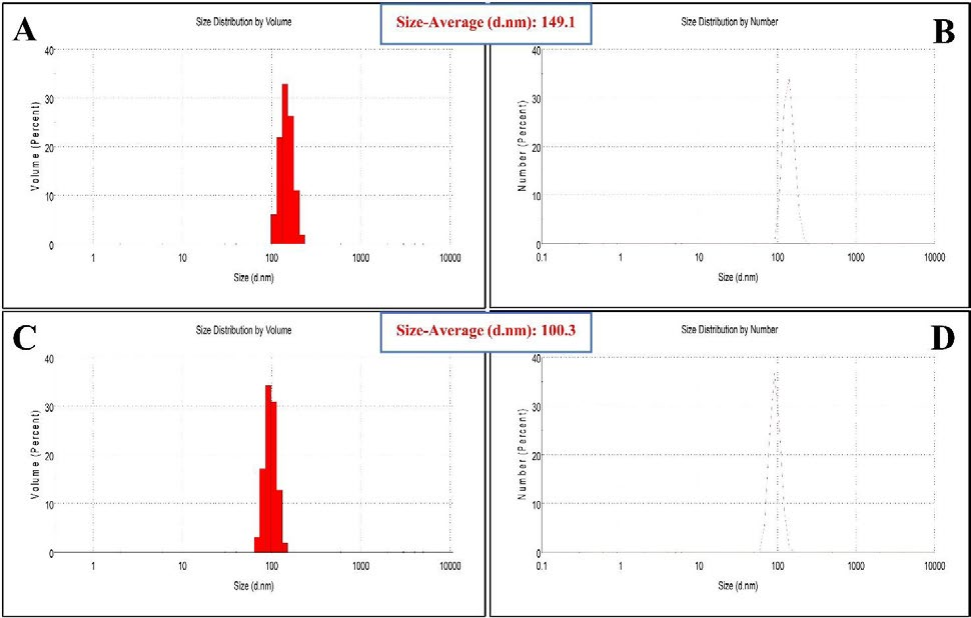
Size distribution obtained by Zetasizer for P3 (A, B) and P3/E10 nano-complexes (C, D)

**Figure 2:**
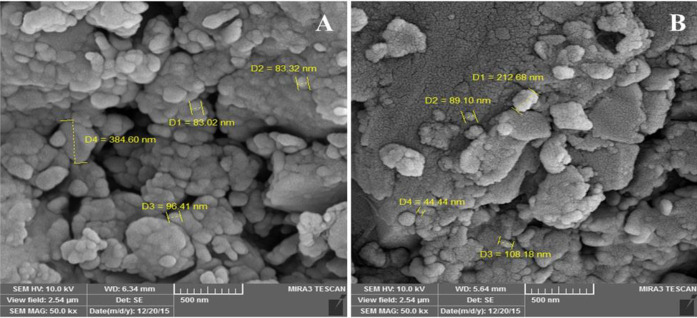
SEM images of P3 (A) and P3/E10 nano-complexes (B)

Zetasizer was used to evaluate the size distribution of obtained peptide (P3) and its nano-complex formed in the presence of E_10_ (P3/E_10_). The average size of P3 was reported to be 149.1 nm (PDI = 0.39) (Figures 1A, B), while the P3/E_10_ nano-complexes were smaller in size, measured to be 100.3 nm (PDI = 0.34) (Figures 1C, D). Based on SEM images, P3 showed spherical structure, while P3/E_10_ nano-complexes were rod-like nanostructures with smaller sizes (Figures 2A, B). 

*Cell viability*

**Figure 3:**
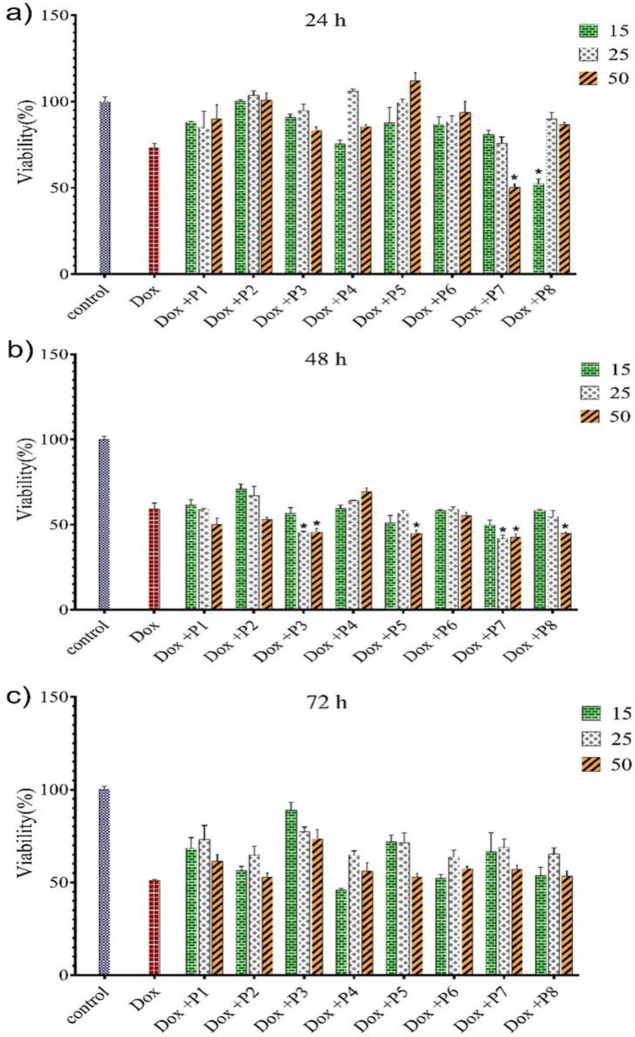
Effects of CPPs on Dox toxicity against A549 Dox-resistant cell line after 24 hr (a), 48 hr (b), and 72 hr (c) exposure at 3 concentration levels including 15, 25, and 50 µM

MTT assay was employed to examine the effects of CPPs on Dox cytotoxicity against A549 Dox-resistant cells at different concentrations including 15, 25, and 50 µM of CPPs. Before the experiment, IC_50_ of Dox was assessed, which was measured to be 20 µM. As shown in Figure 3a, at 15 µM concentration of peptides (P1-P7) and after 24 hr, there was no enhancement in toxicity of Dox in comparison with cells treated with free Dox and control, while P8 significantly reduced the cell viability to 52.5%. 25 µM and 50 µM of P7 also resulted in Dox cytotoxicity. 

After 48 hr, 50 µM of P5, P7, and P8 increased the cytotoxicity of Dox (Figure 3b). After 72 hr, compared with cells treated with free Dox and control, there was no meaningful change in the cell viability, except for the group treated with 15 µM of P4-Dox (Figure 3c).

*Cellular uptake*

**Figure 4:**
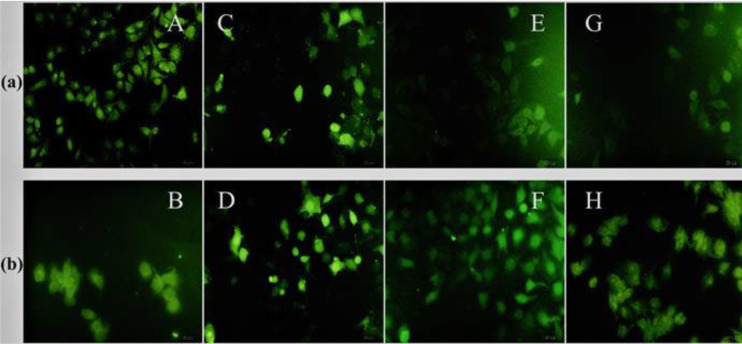
Fluorescent Images of row (a): A (FP7 25 µM), C (FP7 50 µM), E (FP8 25 µM), and G (FP8 50 µM); row (b): B (FP7/E10 25 µM), D (FP7/E10 50 µM), F (FP8/E10 25 µM), and H (FP8/E10 50 µM) on A549 cells

FP7: FAM-labeled P7; FP8: FAM-labeled P8

Fluorescent microscopy was utilized to examine the cellular uptake of 25 and 50 µM FAM-labeled P7 (FP7), FAM-labeled P8 (FP8), and FP7/E_10 _and FP8/E_10_ nano-complexes on A549 cells (Figures 4A, B). Based on results, at 25 µM, FR_10 _showed the highest uptake and its conjugation with E_10_ did not have any effect on its cellular uptake, whereas the nucleus uptake was slightly decreased. While the highest uptake was observed at 50 µM of FP7/E_10_, at both concentrations, FP8/E_10_ illustrated better uptake in comparison with FP8. Generally, arginine-rich peptides had better uptake than those with lysine. Furthermore, they were mostly distributed around the cell nucleus and demonstrated homogenous staining through the cell structure.

**Figure 5:**
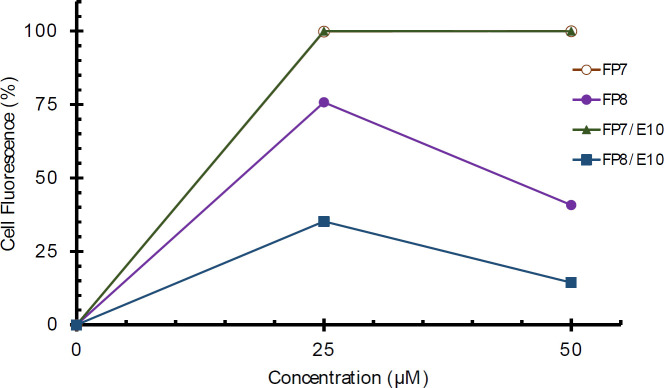
Cellular uptake of FP7, FP8, and FP7/E10, and FP8/E10 nano-complexes by A549 cells. The uptake was measured as the relative fraction of positive cells (%) from flowcytometry analysis of all living cells positive for the fluorophore

FP7: FAM-labeled P7; FP8: FAM-labeled P8

Flow cytometry was used to quantitatively assess the cellular uptake of FR_10, _FK_10_, and their nano-complexes at the same concentrations. FP7 and FP7/E_10_ showed similar behavior with high efficiency in cell entrance, while FP8 and FP8/E_10_ showed poor cellular uptake. Furthermore, by increasing the concentration from 25 to 50 µM, the cellular uptake of FP8 was decreased (Figure 5).

**Figure 6:**
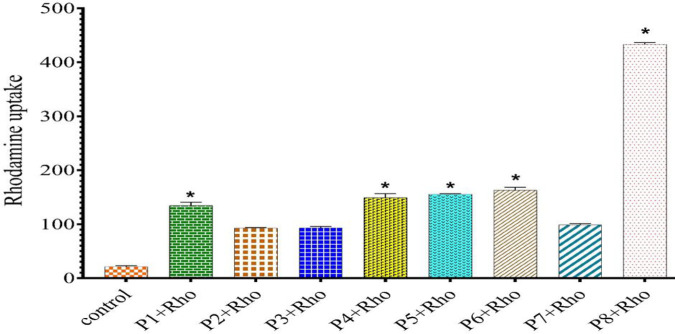
Rhodamine123 accumulation in the presence of CPPs after 48 hr incubation

To investigate how the CPPs impact P-gp function, rhodamine123 accumulation assay was carried out (excitation and emission at 485 and 530 nm, respectively) for each sample (Figure 6). According to our results and compared with control, all CPPs could successfully increase the rhodamine123 uptake. P4, P5, and P6 had a practically similar impact on the rhodamine123 accumulation, while P8 had a significant effect on P-gp. On the other hand, compared with similar behavior of both P3 and P2, P1 led to higher accumulation. P7 was in competition with block and alternative sequences of peptides containing tryptophan and lysine.

*ABCB1 expression assay*

**Figure 7:**
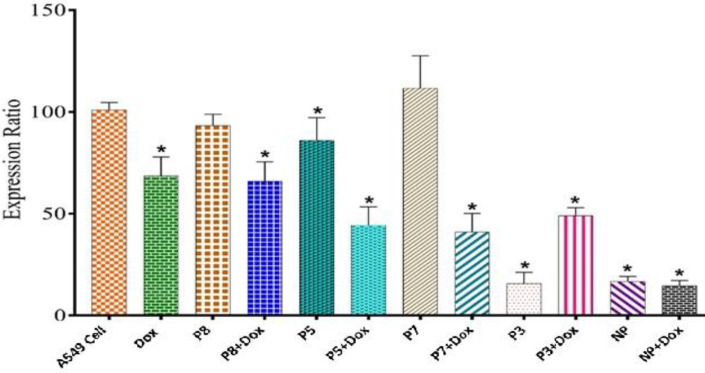
ABCB1 expression level in A549 cell line after 48 hr of treatment with different peptides and peptide-Dox formulations

ABCB1 expression in the A549 cell line was assessed using real-time PCR. In this regard, cells were treated with the same concentrations of peptides and Dox, then the RNA was extracted and its cDNA was synthesized and normalized. Compared with controls and among all the designed CPPs, P3 had the most significant inhibitory effect on the ABCB1 gene expression, however, the Dox inhibitory behavior was not improved. Furthermore, P5-Dox and P7-Dox, significantly decreased gene expression (Figure 7). Moreover, the P5/E_10_ nano-complexes were employed to investigate their effects on gene expression. As a result, they either solely or in the presence of Dox, considerably reduced the expression level. 

## Discussion

In the present study, the morphology, size, and cellular uptake of obtained peptides were assessed by SEM, Zetasizer and fluorescence microscopy, and flow cytometry, respectively. Based on SEM images and Zetasizer analysis, the addition of E_10_ to peptides resulted in tangible changes to their size and morphology. SEM images of P3/E_10_ nano-complexes represented the creation of rod-like nanostructures that were connected to each other. The formation of these structures could be explained by the presence of electrostatic attraction (salt bridges) formed between cationic arginine/lysine residues from two different peptide chains and polyanionic polyglutamate (E10) residues (38).

There are two major mechanisms introduced for cellular uptake of CPPs including endocytosis and direct translocation (39, 40). Our results indicated higher uptake efficacy of P7 compared with P8, which might be due to the fact that the cell surface proteoglycans promote the uptake of P7 through hydrophobic interactions, while P8 experiences electrostatic interactions. In the absence of proteoglycans, arginine-rich peptides with 5 to 15 residues use other pathways to sufficiently enter the cells (41), which justifies the efficient uptake of P7. It is worth noting that, guanidinium residue is necessary for faster uptake of synthetic arginine-rich peptides (42) which is in contrast with the natural CPPs like TAT (Trans Activator of Transcription). The main mechanism for cellular uptake of CPPs is not fully understood, but studies with fluorescent microscopy and flow cytometry support the direct translocation of arginine-rich peptides into cells (35, 36, 43-49). To further study the cellular uptake of obtained peptides, fixed cells led to the nuclear distribution of peptides while in unfixed cells, they mostly distributed in cytoplasmic parts. Furthermore, we could not distinguish the amount of internalized and membrane-attached peptides by flow cytometry. In the recent study and based on the results acquired from confocal microscopy, polycationic peptides like poly K along with poly R were not taken efficiently by cells (50), while integrating poly glutamates like E_8_ into the structure of peptides could enhance the cellular uptake (45, 47), however, in the present study, E_10_ could not lead to dramatic changes in the cellular uptake of P7. Moreover, P8 had higher uptake compared with P8/E_10_ nano-complexes in different concentrations. On the contrary, rhodamine123 accumulation studies suggested that the lysine-rich peptides increase rhodamine123 accumulation. Dox is a hydrophobic anticancer drug with short retention time within the cells due to the robust activity of efflux pumps in cancer cells. This study represents P3, P5, P7, and P8 as promising CPPs that could increase the Dox retention time and its cytotoxic behavior in A549 Dox-resistant cells probably due to inhibition of negatively charged P-gp efflux pumps. Furthermore, CPPs can also interact with P-gp through forming hydrogen bonds and acting as its substrates (30), which extends the cellular retention time of Dox. Based on our observations, the main mechanism for cellular uptake of designed CPPs was direct translocation, which did not lead them to endosomal entrapment and degradation and probably this was the main reason for improved cytotoxic behavior of Dox at higher concentrations of CPPs. Another study used cyclic [W(WR)_4_]-Dox and linear [W(WR)_4_]-Dox (30) to increase the cellular uptake of Dox by inhibiting the efflux pumps. The cyclic structure could exhibit higher efficacy compared with the linear one (51). Based on the same mechanism and using the cationic peptide (NK-2) at 5 µM concentration, Dox resistance was reversed in drug-resistant lung cancer cell lines (52). In another study, R_8_-Dox complexes were prepared to evaluate their anticancer activity compared with free Dox. At 10 µM concentration and after 24 hr, R_8_ improved the toxicity of Dox, while their separate employment at the same concentration did not show any significant cytotoxicity. This was possibly due to the fact that R_8_ had high permeability and affinity for cellular DNA and RNA, resulting in Dox affinity towards targeted cells (53). In the present study, P7 also increased the Dox toxicity at concentrations of 25 and 50 µM. Furthermore, real-time PCR was applied to see the ABCB1 expression in different samples, which only shows the gene expression level and it was not clear whether the amount of the protein was increased, indicating the requirement for Western blot for complete studies. P3 could successfully decrease ABCB1 expression, while P5 unexpectedly enhanced its expression level. In the presence of Dox, the designed CPPs except P3 could decrease the gene expression level. On the other hand, P-gp expressing cells show cytoplasmic alkalization and extracellular acidification by catalyzing ATP-dependent proton extrusion (52). In fact, energetic alterations of cell membrane depolarization, low transmembrane potential, and low extracellular pH as well as perturbations in cellular ion transport are considered as characteristics of MDR cancer cells. These are favorable for cationic peptides to act against P-gp. Duan *et al.* evaluated the antitumor ability of paclitaxel (PTX)-conjugated TAT and LMWP (low molecular weight protamine) in comparison with free PTX on A549 cancer cells. Results indicated that PTX-LMWP has the potential for inhibiting tumor growth and considered a promising candidate to treat drug-resistant lung cancer (54). To bypass P-gp and overcome MDR cells, a hybrid CPP, comprising TAT and a drug binding motif (DBM) has been developed to deliver Dox into the K562 leukemia cell line and its P-gp overexpressing subline KD30. In this study, the endocytic cellular uptake of Dox was significantly enhanced in both cell lines, and it was supported that, this combination reduced the P-gp-mediated drug efflux (55).

**Table 1:** The sequence of used primers and their important properties

*Primer*	*Sequence*	*T*_m_ * (* *°* *C)*	*PCR product (bp)*
**β-actin**	Forward:5CTCACCATGGATGATGATATCGC Reverse:5AGGAATCCTTCTGACCCATGC	62.9 61.3	23 21
**ABCB1**	Forward:5GAGAGATCCTCACCAAGCGG Reverse:5ATCATTGGCGAGCCTGGTAG	62.5 60.5	20 20

**Table 2:** The sequence and molecular properties of synthesized peptides

***Peptide***	*C-Sequence-N*	*Chemical formula*	*Molecular weight (g/mol)*	*Isoelectric point*	*Net Charge at pH=7*	*Extinction coefficient M* ^-1^ *C* ^-1^	*Approximately* *volume A* ^3^
**P1**	COOH-[WR]_4_-NH_2_	C_68_H_90_N_24_O_9_	1387.62	pH 12.88	4	22760	1679
**P2**	COOH-[WK]_4_-NH_2_	C_68_H_90_N_16_O_9_	1275.56	pH 11.01	4	22760	1544
**P3**	COOH-[W_4_K_4_]-NH_2_	C_68_H_90_N_16_O_9_	1275.56	pH 11.01	4	22760	1544
**P4**	COOH-[W_4_R_4_]-NH_2_	C_68_H_90_N_24_O_9_	1387.62	pH 12.88	4	22760	1679
**P5**	COOH-[WR]_3_-QGR-NH_2_	C_64_H_91_N_25_O_11_	1386.59	pH 12.88	4	17070	1678
**P6**	COOH-QGR-[WK]_3_-NH_2_	C_64_H_91_N_19_O_11_	1302.55	pH 11.73	4	17070	1577
**P7**	COOH-R_10_-NH_2_	C_60_H_122_N_40_O_11_	1579.9	pH 13.35	10	0	1912
**P8**	COOH-K_10_-NH_2_	C_60_H_122_N_20_O_11_	1299.76	pH 11.46	10	0	1573
**P9**	COOH-QGR-[WR]_3_-NH_2_	C_64_H_91_N_25_O_11_	1386.59	pH 12.88	4	17070	1678
**P10**	COOH-WRWQGRWRW-NH_2_	C_69_H_89_N_23_O_11_	1416.61	pH 12.7	3	22760	1715
**E**_10_	COOH-E_10_-NH_2_	C_50_H_72_N_10_O_31_	1309.17	pH 2.73	-10	0	1584

** Note: [E: Glutamic Acid, W: Tryptophan, R: Arginine, Q: Glutamine, G: Glycine, K: lysine]

CPPs: cell-penetrating peptides

ABCB1: ATP-binding cassette B1

## Conclusion

Results showed that employment of CPPs with chemotherapeutics could improve chemotherapy efficiency *via* bypassing P-gp and overcoming the MDR phenomenon. Four synthesized peptides including P3, P5, P7, and P8 could successfully increase the accumulation of Dox within the drug-resistant A549 cells. PCR studies also proved the positive effects of designed peptides, in which they improved the Dox cytotoxicity by decreasing the efflux pumps gene expression. Finally, mentioned peptides are presented as effective agents to be applied with chemotherapeutics to enhance the survival rate of patients suffering from cancer. 
